# Development of a Sphingosylphosphorylcholine Detection System Using RNA Aptamers

**DOI:** 10.3390/molecules15085742

**Published:** 2010-08-20

**Authors:** Katsunori Horii, Kazuya Omi, Yoshihito Yoshida, Yuka Imai, Nobuya Sakai, Asako Oka, Hiromi Masuda, Makio Furuichi, Tetsuji Tanimoto, Iwao Waga

**Affiliations:** 1 VALWAY Technology Center, NEC Soft, Ltd., Tokyo 136-8627, Japan; E-Mails: yoshida-y@mxb.nes.nec.co.jp (Y.Y.); sakai-nobuya@zx.mxy.nes.nec.co.jp (N.S.); masuda-hiromi@mxg.nes.nec.co.jp (H.M.); furuichi@mxb.nes.nec.co.jp (M.F.); waga-iwao@mxc.nes.nec.co.jp (I.W.); 2 Biotechnology Research Group, Fundamental Research Department, Fujirebio Inc., Tokyo 192-0031, Japan; E-Mails: ky-omi@fujirebio.co.jp (K.O.); yk-imai@fujirebio.co.jp (Y.I.); ak-oka@fujirebio.co.jp (A.O.); tj-tanimoto@fujirebio.co.jp (T.T.)

**Keywords:** aptamer, enzyme-linked aptamer assay, sphingosine 1-phosphate, sphingosylphosphorylcholine, systematic evolution of ligands by exponential enrichment

## Abstract

Sphingosylphosphorylcholine (SPC) is a lysosphingolipid that exerts multiple functions, including acting as a spasmogen, as a mitogenic factor for various types of cells, and sometimes as an inflammatory mediator. Currently, liquid chromatography/tandem mass spectrometry (LC/MS/MS) is used for the quantitation of SPC. However, because of the complicated procedures required it may not be cost effective, hampering its regular usage in a routine practical SPC monitoring. In this report, we have generated RNA aptamers that bind to SPC with high affinity using an *in vitro* selection procedure and developed an enzyme-linked aptamer assay system using the minimized SPC aptamer that can successfully distinguish SPC from the structurally related sphingosine 1-phosphate (S1P). This is the first case of the Systematic Evolution of Ligands by EXponential enrichment (SELEX) process being performed with a lysosphingolipid. The SPC aptamers would be valuable tools for the development of aptamer-based medical diagnosis and for elucidating the biological role of SPC.

## 1. Introduction

**Figure 1:**
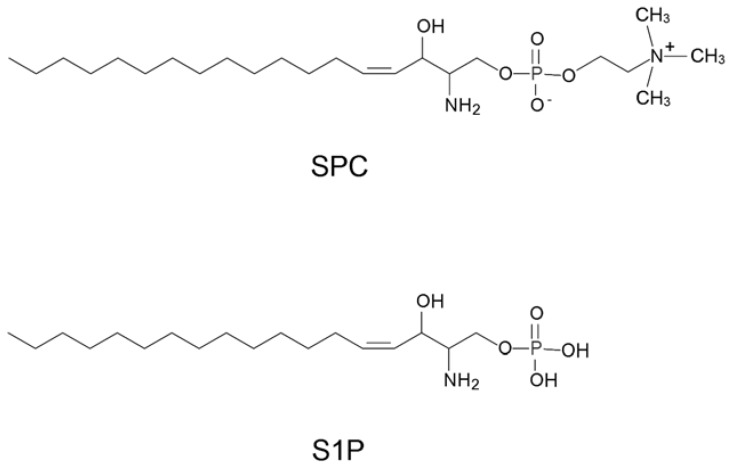
Chemical structures of SPC and S1P.

Sphingosylphosphorylcholine (SPC) is produced from sphingomyelin by *N*-deacylase [1] and has a structure related to sphingosine 1-phosphate (S1P, Figure 1), which is one of the major lipid mediators bound to G-protein-coupled receptors and evokes signal responses [2]. SPC plays various functional roles [3], such as a mitogen in several types of cells including endothelial cells, fibroblasts, keratinocytes, and vascular smooth muscle cells *in vitro* [4,5,6], and SPC also participates in wound healing by stimulating cell proliferation [7] and functions as an inflammatory mediator *in vivo* [8]. Interestingly, SPC and S1P evoke similar cell responses via distinct mitogen-activated protein kinases [9], although the receptor for SPC remains unclear. It is also known that SPC is significantly upregulated in the stratum corneum of patients with atopic dermatitis as a result of a dramatic increase in sphingomyelin deacylase activity [10].

Recently, Kurokawa *et al.* suggested the possibility that SPC is related to cerebral vasospasms, which are a major complication in patients with subarachnoid hemorrhages (SAHs) [11]. They showed that the concentration of SPC in human cerebrospinal fluid (CSF) is elevated after SAH to about 30 nM, which is significantly higher than that in normal CSF (1.6 nM), but lower than that in normal plasma and serum (50 and 130 nM, respectively) [12]. They also demonstrated that SPC in CSF is rapidly diluted in the canine model, suggesting that much higher levels of SPC are actually released into CSF after SAH. SPC is an upstream mediator of Rho-kinase [13], whose activity is thought to contribute to the severe arterial narrowing observed in cerebral vasospasm after SAH [14].

For quantitative and specific analysis of sphingolipids, currently the only technique available is liquid chromatography coupled to tandem mass spectrometry (LC/MS/MS), as other techniques are inadequate for evaluating the diversity of sphingolipids [11,15,16]. However, this method requires an expensive instrument, resulting in high assay costs in addition to routinely complicated procedures.

In this study, we demonstrated first that RNA aptamers bind to SPC with high affinity and selectivity. Aptamers were isolated from RNA pools using the iterative Systematic Evolution of Ligands by EXponential enrichment (SELEX) process [17,18]. The selected aptamers were characterized for binding affinity using surface plasmon resonance (SPR) and truncated for cost-effective production. We succeeded in minimization of the length to 33 nucleotides with sub-micromolar dissociation constants. An enzyme-linked aptamer assay (ELAA) with the minimized aptamer was established and used to demonstrate that this aptamer has the potential to distinguish between SPC and the structurally related S1P. These aptamers could be useful for the development of quantitative, selective, and cost-effective detection of SPC.

## 2. Results and Discussion

### 2.1. Selection of SPC binding aptamers

**Figure 2:**
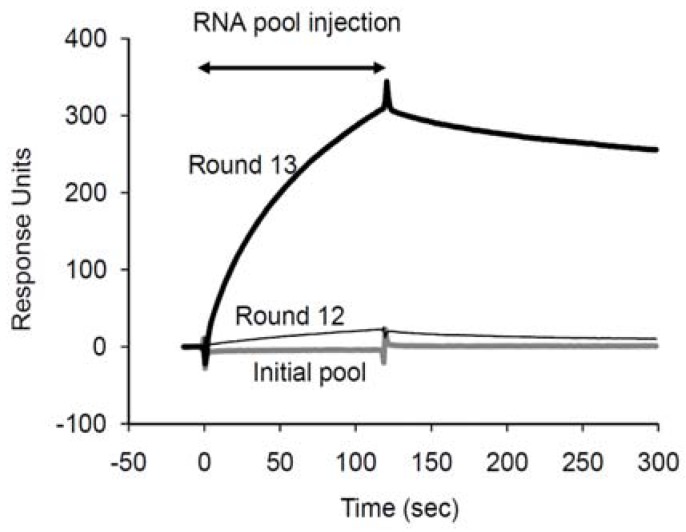
Binding analysis of the selection pools using SPR. The interactions between the selection pools (round 0, 12, 13) and the immobilized SPC on a SA sensor chip were investigated. The concentration of each pool was adjusted to 2 µM in the selection buffer.

Twelve rounds of traditional *in vitro* selection with SPC beads were performed to isolate aptamers that bound to SPC. After round 12, the SPC binding assay of the pool using SPR indicated favorable binding properties to SPC (Figure 2). 

However, 44 clones derived from the round showed low sequence similarity (data not shown), suggesting that even though the SPC-binding aptamers are enriched in the pool, many RNA molecules that may bind to the agarose resins or linker are still remaining. Hence, a further round of selection using a SPR sensor chip with the Biacore 3000 instrument instead of the resins was performed to reduce the non-specific binding to resins. Five-micromolar RNA pool was injected over the immobilized biotinylated SPC on a SA-coated sensor chip (Biacore, GE Healthcare, Uppsala, Sweden). Unbound and weakly bound RNA molecules were washed with the selection buffer containing Tween-20 (SBT: 20 mM HEPES, pH 7.4, 150 mM NaCl, 5 mM MgCl_2_, 5 mM KCl, 0.005% Tween-20). The advantage of this method is the ability to control washing stringency by monitoring the SPR signal during the process. The bound RNA molecules were recovered by adding EDTA to chelate magnesium ions. The binding signal of the final recovered pool (round 13) was significantly increased more than 10-fold compared with that of the previous round (Figure 2), suggesting that the population of the SPC aptamers was dramatically increased in the final pool. Sequence alignment of 85 clones from the final pool revealed that they were classified into 9 groups differing by one base within each group and the other independent clones. The RNA sequences of the major groups (C1–C9) are shown in Table 1. The most enriched clones belonged to C1 and accounted for approximately 14% of the final pool. A consensus sequence, 5′-CRUURUUG-3′, was observed in the randomized region of 9 groups. Interestingly, this sequence was found adjacent to the conserved forward primer region in all groups except for C8, implying that the primer region may participate in SPC recognition.

**Table 1:** Sequences of the SPC aptamers (C1–C9) and their truncated variants. Lower case letters indicate nucleotides derived from the fixed sequences. The conserved hairpin motif is underlined.

Clone ID	Frequency	Sequence
C1	12/85	gggaauggauccacaucuacgaauucUACCGUUAUUGGUGUCACCGAAGAUGUUAuucacugcagacuugacgaagcuu (79 mer)
C2	7/85	gggaauggauccacaucuacgaauucUUCCGUUAUUGGAGCCAAGUCGUAUCCCGAuucacugcagacuugacgaagcuu (80 mer)
C3	4/85	gggaauggauccacaucuacgaauucUACCGUUAUUGGAGCACGCGUAGUAUGGUUuucacugcagacuugacgaagcuu (80 mer)
C4	3/85	gggaauggauccacaucuacgaauucUACCGUUAUUGGUGUAACGUAAUUGUGAuucacugcagacuugacgaagcuu (78 mer)
C5	2/85	gggaauggauccacaucuacgaauucUACCAUUAUUGGAGCUGUCGAUUUGCUGGAuucacugcagacuugacgaagcuu (80 mer)
C6	2/85	gggaauggauccacaucuacgaauucUCCCGUUAUUGGAGUCACGCGUAGUCCUCCuucacugcagacuugacgaagcuu (80 mer)
C7	2/85	gggaauggauccacaucuacgaauucUACCGUUGUUGGAGACGCUUAGAUGUCCGAuucacugcagacuugacgaagcuu (80 mer)
C8	2/85	gggaauggauccacaucuacgaauucGCAUUGUCCGCACGCAAAGCAUUAUUGUGAuucacugcagacuugacgaagcuu (80 mer)
C9	2/85	gggaauggauccacaucuacgaauucGUCCGUUAUUGGCGCCAGCGUACAUGCGGGuucacugcagacuugacgaagcuu (80 mer)
m009		GggauccacaucuacgaauucUACCGUUAUUGGUGUCACCGAAGAUGUUAuucC (54 mer)
m010		GGaucuacgaauucUACCGUUAUUGGUGUCACCGAAGAUCC (41 mer)
m011		GggauccacaucuacgaauucUUCCGUUAUUGGAGCCAAGUCGUAUCCCGAuucC (55 mer)
m012		GGcgaauucUUCCGUUAUUGGAGCCAAGUCGCC (33 mer)

### 2.2. Binding assay of the selected aptamer candidates using SPR

**Figure 3:**
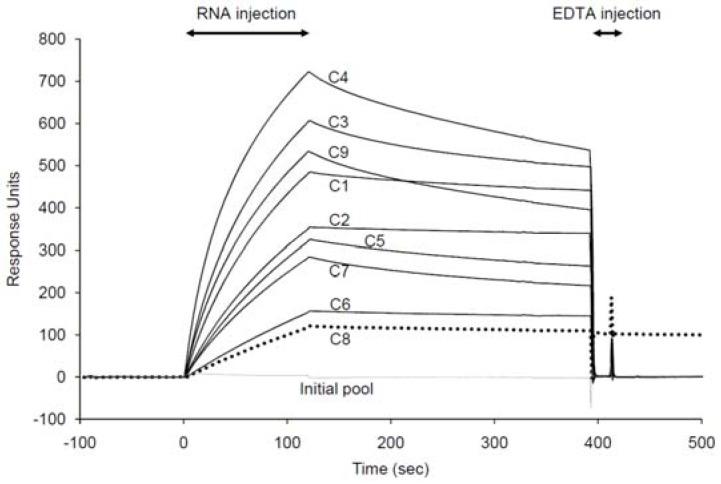
Binding of the enriched clones (C1–C9) to the immobilized SPC. Forty microliters of each 500-nM aptamer in the selection buffer with 0.005% Tween 20 was injected individually at a flow rate of 20 µL/min. All clones bound to SPC were removed completely except for C8 (dotted line) by adding 20 µL of 10 mM EDTA at a flow rate of 60 µL/min.

The binding affinities of the enriched clones (C1–C9) to SPC were analyzed using SPR. For all clones, binding signals were obtained with various amounts (Figure 3). All bound clones except for C8 were released completely by adding 10 mM EDTA (Figure 3), suggesting that magnesium ions are required for binding of the clones to SPC. On the other hand, divalent cations were not essential for binding of C8 to SPC. Further characterizations were performed for C1, C2, and C8 because these clones had slow off-rates as compared with the others. Kinetic parameters, calculated from the sensorgrams by fitting with a 1:1 (Langmuir) binding model, are listed in Table 2. The dissociation constants (*K*_D_) of these clones indicated that they have high affinities under 100 nM.

**Table 2:** Kinetic parameters of the SPC aptamers at 25 °C.

Aptamer	*k*_a_ (10^4^/Ms)	*k*_d_ (10^-4^/s)	*K*_D_ (nM)
C1	2.04 ± 0.01	4.27 ± 0.03	20.9 ± 0.19
C2	1.41 ± 0.01	4.67 ± 0.01	33.2 ± 0.19
C8	0.59 ± 0.02	5.36 ± 0.02	90.4 ± 2.35
m009	1.24 ± 0.33	23.1 ± 0.08	186 ± 5.05
m010	1.17 ± 0.33	29.5 ± 0.09	252 ± 7.04
m011	1.13 ± 0.25	24.9 ± 0.06	221 ± 4.84
m012	1.11 ± 0.17	21.8 ± 0.04	196 ± 2.98

### 2.3. Secondary structure prediction and minimization of the SPC aptamers

**Figure 4:**
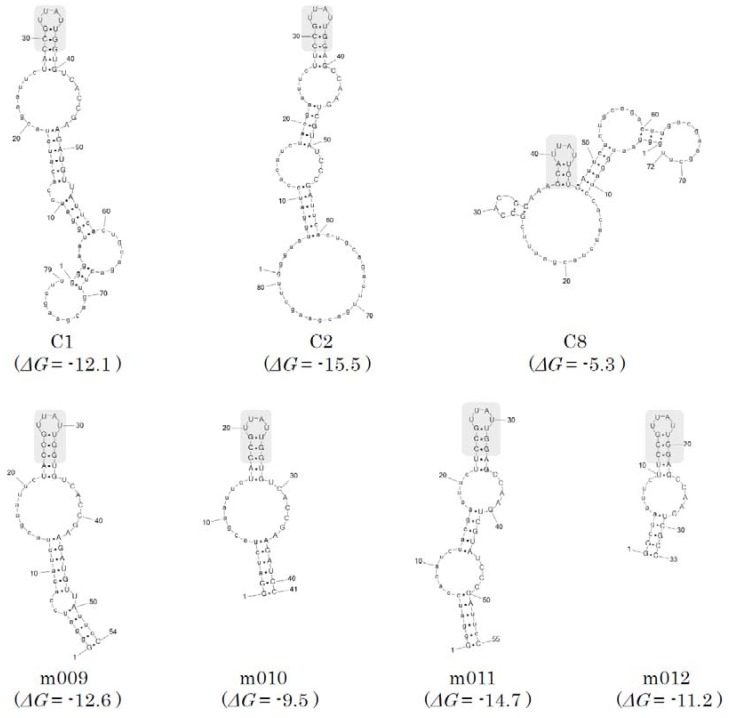
Predicted secondary structures of the SPC aptamers. The estimated stabilities, *ΔG* (kcal/mol), are shown in parentheses. The conserved hairpin structure is highlighted with gray backgrounds. All RNA structures were visualized using the PseudoViewer3 program [20].

**Figure 5:**
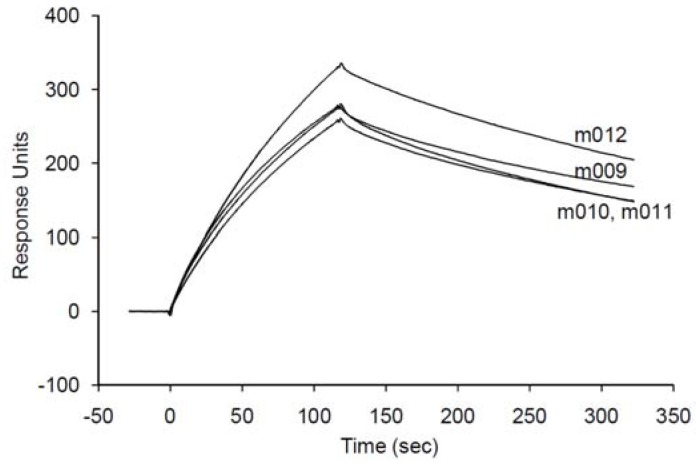
The overlaid SPR sensorgrams of the truncated variants (m009–m012) derived from the initially determined clones (C1 and C2). The measurements were performed under the same conditions as for Figure 3.

For each selected aptamer, one of the stable structures associated with small free energy was predicted using the ValFold program (Figure 4) that adopts empirical free energy parameters [19]. A unique stem-loop conformation was conserved among the aptamers, suggesting that this region is critical for binding to SPC. To produce SPC aptamers by chemical synthesis cost-effectively, 4 truncated forms (m009–m012) derived from C1 and C2 were designed (Figure 4). SPR binding experiments for these truncated forms showed that although the affinity was decreased by one order due to their fast off rates compared with those of the original aptamers, they all successfully bound to SPC with good affinity (*K*_D_ = ~200 nM) (Figure 5 and Table 2). These results suggest that the predicted SPC binding sites and secondary structures are quite reliable.

Previous studies on RNA aptamers against phospholipids bilayers [21], [22], amphiphilic phosphoglycolipids [23], and aliphatic amino acids [24] revealed that G-rich motifs or G/U motifs in RNA were important for the binding of hydrophobic targets. However, these G-rich motifs were not found in SPC aptamers, suggesting that the binding mechanism of the aptamers to SPC may differ from that against hydrophobic targets.

### 2.4. Validation of an SPC assay system based on the minimized aptamer

**Figure 6:**
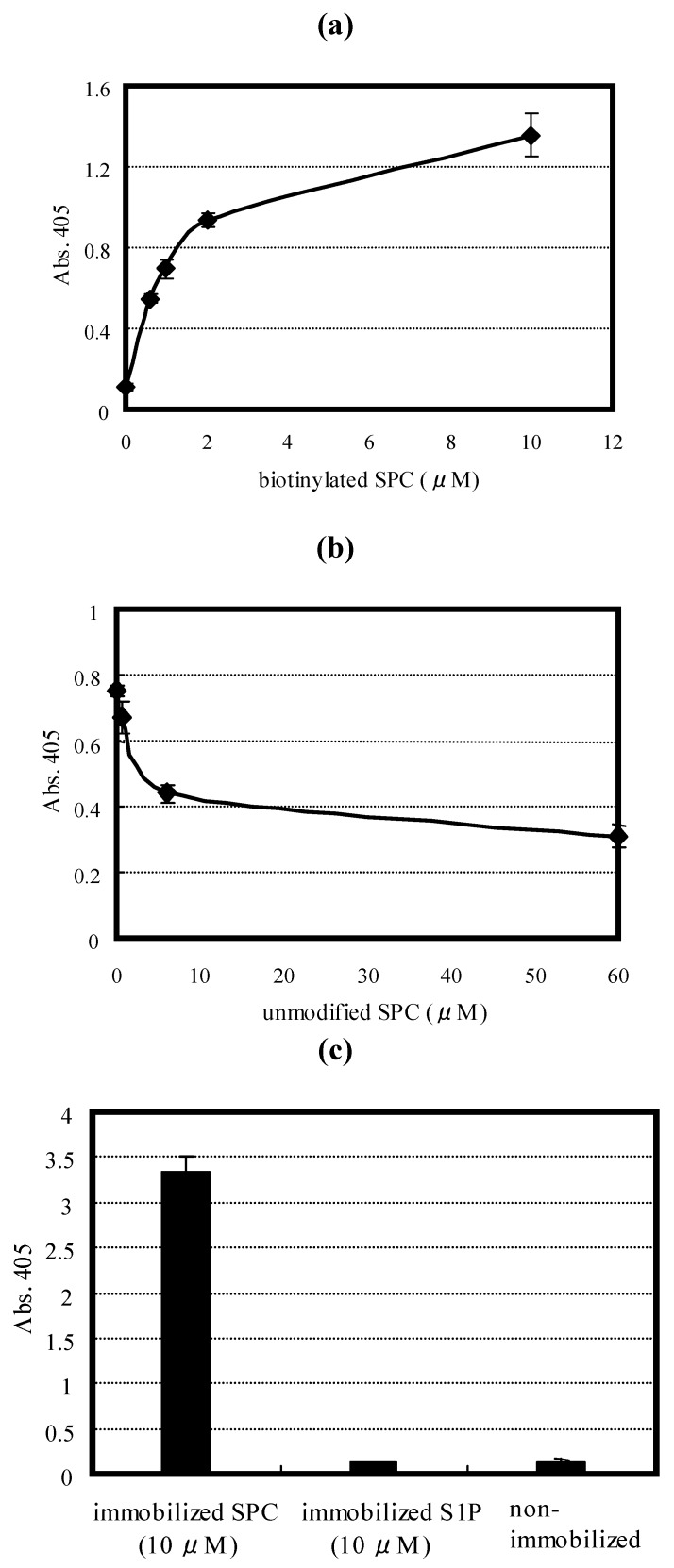
ELAA. (a) Sensitivity of the m012 aptamer for biotinylated SPC. The amount of biotinylated SPC captured by immobilized m012 was shown as the absorbance at 405 nm. The experiment was repeated three times with each data point measured in duplicates, and a representative result is shown. (b) Competitive ELAA using m012 and biotinylated and unmodified SPC. The biotinylated and serially diluted unmodified SPC were added to the m012-immobilized plate. The amount of biotinylated SPC captured by the immobilized m012 was shown as the absorbance at 405 nm. The experiment was repeated three times with each data point measured in duplicates, and a representative result is shown. (c) Specificity of the m012 aptamer for SPC and S1P. The amount of the biotinylated m012 reacted with the immobilized biotinylated SPC and S1P was shown as the absorbance at 405 nm. The experiment was repeated three times in triplicates, and a representative result is shown. Error bars show standard deviations.

An ELAA was applied to the SPC assay system for which the m012 aptamer was used because of its short length and better affinity in the truncated forms. The immobilized m012 aptamer on a plate was demonstrated to capture the biotinylated SPC in a dose-dependent manner (Figure 6a), indicating that m012 is applicable to an ELAA format. The sensitivity of m012 to the biotinylated SPC was at least > 600 nM. The reactivity of m012 to unmodified native SPC was also examined using the competitive ELAA. The ELAA values decreased as the amounts of native SPC mixed with the biotinylated SPC increased (Figure 6b), suggesting that m012 has an ability to bind to native SPC as well as biotinylated SPC.

**Figure 7:**
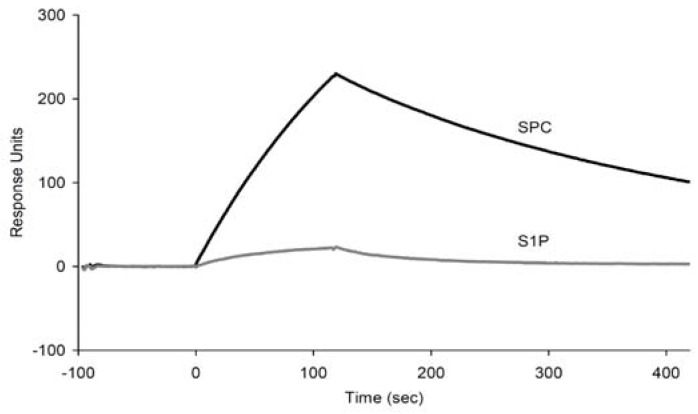
Bindinganalysis of the m012 aptamer to SPC and S1P using SPR. The biotinylated SPC and S1P were immobilized on each flow cell at similar amounts (~250 RU). 400 nM aptamer was injected over the ligands for 2 min.

To evaluate the specificity of the aptamer, the binding experiments using ELAA and SPR were performed with S1P, which is a structurally related signaling molecule to SPC and naturally occurs in human serum at a concentration of 130 nM [25]. The ELAA experiment showed that the immobilized S1P was undetectable with m012 at a concentration of 10 µM (Figure 6c). The SPR measurement also demonstrated that the amount of m012 bound to S1P (400 nM) was quite low compared with that to SPC (Figure 7). These data show that m012 can bind to SPC selectively without cross-reactivity to S1P.

Compared with two chemical structures, SPC is more positively charged than S1P due to the choline moiety (Figure 1). Therefore, it might be suspected that the aptamers bind to SPC with nonspecific electrostatic interactions by positively charged choline of SPC and negatively charged backbone of RNA. However, most RNA molecules in the randomized pool did not interact to SPC at all in the binding buffer (Figure 2), suggesting that such nonspecific interactions are quite rare. The bound aptamers to SPC were released by removal of magnesium ions with a chelating agent (Figure 3), indicating that some specific spatial RNA conformations with magnesium ions are required to recognize SPC. In addition, a mutant of the C1 aptamer, which was substituted U with C in the conserved hairpin-loop region, UUAUU (the underline indicates the mutation site), exhibited much lower binding to SPC (data not shown), though the predicted secondary structure of the mutant was identical to that of the C1 aptamer. This result demonstrates that the uracil base of the mutation site in the hairpin-loop is critical for the binding and might contact to SPC directly. Thus, the aptamers would form a proper three dimensional structure, recognize SPC specifically and distinguish between SPC and S1P.

## 3. Experimental

### 3.1. Reagents

SPC (CAS No. 1670-26-4) and its analog were labeled at the terminus of the alkyl chain with biotin groups that were purchased from BIOMOL Research laboratories (Plymouth Meeting, PA) and Avanti Polar Lipids (Alabaster, AL), respectively. S1P (CAS No. 26993-30-6) and its biotinylated analog were purchased from Avanti Polar Lipids. Streptavidin (SA) and NeutrAvidin® (NA) Plus Ultralink® resins were purchased from Thermo Fisher Scientific/Pierce Biotechnology (Rockford, IL) and used to immobilize the biotinylated SPC for the SELEX procedure. A DNA template and primers (OPC grade) were obtained from Hokkaido System Science (Hokkaido, Japan). Ex Taq™ DNA Polymerase and Transcriptor Reverse Transcriptase were purchased from TaKaRa Bio (Ohtsu, Japan) and Roche Applied Science (Indianapolis, IN), respectively. RNAs were prepared by *in vitro* transcription (IVT) using the AmpliScribe™ Transcription kit (Epicentre Technologies, Madison, WI) or by chemical synthesis (Japan Bio Services, Saitama, Japan). The biotinylated aptamer was produced by chemical synthesis and purified by HPLC. All cloning was performed with the pGEM®-T Easy vector system (PROMEGA, Madison, WI). Mach1™ competent cells (Invitrogen, San Diego, CA) were used for transformation. DNA sequencing samples were prepared with the illustra TempliPhi™ DNA sequencing template amplification kits (GE Healthcare, Piscataway, NJ) and analyzed on the ABI PRISM® 310 Genetic Analyzer (Applied Biosystems, Foster City, CA). 

An initial RNA pool, 5'-GGG AAU GGA UCC ACA UCU ACG AAU UC - N30 - UUC ACU GCA GA CUU GAC GAA GC-3′, was generated from the double-stranded DNA pool containing 30 random nucleotides by IVT with T7 RNA polymerase [26]. All RNA pools were purified prior to selection by denaturing polyacrylamide gel electrophoresis.

### 3.2. SELEX

The biotinylated SPC was immobilized on SA- and NA-coated resins to segregate SPC-binding molecules from the non-binding molecules. These two resins were used by turns for each round of selection to remove SA- and NA-specific binders. All RNA pools were heated at 95 °C for 3 min and cooled down gradually to room temperature in the selection buffer (SB: 20 mM HEPES, pH 7.4, 150 mM NaCl, 5 mM MgCl_2_, 5 mM KCl) to generate some stable conformations before mixing with the SPC-coated resins. 

The selection of RNA aptamers was performed starting from 750 pmol of RNAs (~10^14^ molecules) in 150 µL of SB. Binding reactions were performed by adding 150 µL of SPC-coated resins to the RNA pool in a Micro Bio-spin Chromatography Column (BIO-RAD, Hercules, CA) for 3 hours at room temperature. Unbound molecules were removed by spinning down and washing with six column volumes of SB. The SPC-binding molecules were collected by mixing with three column volumes of 7 M urea in 20 mM HEPES (pH 7.4) at 90 °C for 5 min. After removal of urea from the collected fraction by EtOH precipitation, reverse transcription (RT) and PCR were performed according to the manufacturer’s instructions. To avoid PCR artifacts due to over cycles, the cycles were determined by monitoring the amplification with SYBR Green I dyes (TaKaRa, Ohtsu, Japan) using the 7300 Real Time PCR System (Applied Biosystems, Foster City, CA). The PCR product was purified by EtOH precipitation. The RNA pool for the subsequent round of selection was prepared from the purified PCR product by IVT. After 2 rounds of selection, a pre-selection (negative selection) using SPC-uncoated SA or NA resins was introduced prior to selection to reduce non-specific resin binding events. The selection conditions for each round are summarized in Table 3. 

**Table 3:** Summary of the SELEX experiment.

Round	RNA (pmol)	Resins (µL)	Incubation time (min)	Total wash volume (mL)	Pre-selection resins (uL)
1	750	SA 150	180	0.9	0
2	225	NA 150	30	1.5	0
3	112.5	SA 150	30	1.5	150
4	45	NA 150	30	1.5	150
5	15	SA 75	30	1.125	112.5
6	7.5	NA 50	30	1	100
7	1.25	SA 25	30	0.625	50
8	0.625	NA 25	30	0.625	50
9	0.625	SA 25	30	0.625	100
10	0.5	NA 25	30	0.75	100
11	0.375	SA 25	30	0.9	100
12	0.25	NA 25	30	0.9	100

The Biacore 3000 system (Biacore, GE Healthcare, Uppsala, Sweden) was adopted for the final selection to remove unfavorable non-specific resin binders completely [27]. Briefly, 100 µL of 5-µM RNA pool in SB was injected over the SPC-immobilized sensor chip at a flow rate of 20 µL/min. Then, SB was allowed to run for 5 min to wash unbound molecules. The RNA molecules bound to SPC were collected by incubating with 10 mM EDTA at 25 °C for 10 min and running the analyte-recovery command “MS_RECOVER.” The recovered RNA molecules in 2 µL of EDTA solution were precipitated by EtOH with a co-precipitant and used for cloning and DNA sequencing after RT-PCR.

The truncated aptamers were designed from the predicted secondary structures and the sequence motifs, which were found from a set of RNA sequence libraries using our program (to be published elsewhere). Nucleotides that may not contribute to structural stability and motifs were deleted from the both termini.

### 3.3. SPR spectroscopy

All SPR analyses were performed by the Biacore 3000 instrument at 25 °C. Each biotinylated ligand was immobilized on a sensor chip SA at a level of ~250 resonance units. SPC binding assays for the RNA pools of each selection round and the isolated clones were performed in SBT. For kinetic experiments, serial two-fold dilutions of the aptamers from 800 nM down to 50 nM in SBT were injected for 2 min, and dissociation was monitored for 10 min at a flow rate of 20 µL/min. The aptamers bound to SPC except clone C8 were removed from the sensor chip by adding 20 µL of 10 mM EDTA at a flow rate of 60 µL/min. In the case of C8 clone, 10 µL of 50 mM NaOH with 500 mM NaCl was added for the regeneration of the sensor chip at a flow rate of 100 µL/min. All SPR experiment data were analyzed using BIAevalution software (Biacore, GE Healthcare, Uppsala, Sweden). The signal of a streptavidin sensor chip without biotinylated ligands was used as a reference, because all selected aptamers were found not to bind to streptavidin at all.

### 3.4. ELAA

The biotinylated m012 aptamer (20 nM) was immobilized on a SA-coated plate (Thermo Fisher Scientific/Nunc, Waltham, MA) at 37 °C for 30 min. A serially diluted solution of the biotinylated SPC was added to the plate and incubated at 25 °C for 60 min. For competitive ELAA, biotinylated (6 μM) and serially diluted unmodified SPC were added to the plate and incubated at 25 °C for 60 min. After three washes with HEPES buffered saline containing magnesium ion (HBS-Mg: 50 mM HEPES, pH7.4, 150 mM NaCl, 5 mM MgCl_2_), SA-horseradish peroxidase (SA-HRP) conjugate was added and incubated at 37 °C for 30 min to detect the biotinylated SPC captured by the immobilized m012 aptamer. After three washes with HBS-Mg buffer, 50 µL of 2,2-azinobis (3-ethylbenzothiazoline-6-sulfonic acid) ammonium salt (ABTS)/H_2_O_2_ was added, and the plate was incubated at 25 °C for 10 min. The reaction was then stopped by the addition of 0.6 M oxalic acid (50 μL per well), and the absorbance at 405 nm of each well was measured.

To examine the specificity of the m012 aptamer, the biotinylated SPC and S1P derivatives were immobilized on an SA-coated plate at 37 °C for 30 min, and the biotinylated m012 aptamer (10 nM) was added and incubated at 25 °C for 60 min. After three washes with HBS-Mg buffer, SA-HRP conjugate was added and incubated at 37 °C for 30 min to detect the biotinylated m012 aptamer reacted with immobilized SPC and S1P. After three washes with HBS-Mg buffer, 50 µL of ABTS/H_2_O_2_ was added, and the plate was incubated at 25 °C for 20 min. The reaction was stopped by the addition of 0.6 M oxalic acid (50 μL per well), and the absorbance at 405 nm of each well was measured.

## 4. Conclusions

RNA aptamers that bind to SPC with good affinity and selectivity were described. This is the first report using the SELEX process to obtained aptamers that work against lipid mediators. As a next step for development of an SPC detection system, the improvement of RNase resistances will be required to measure SPC in samples derived from serum or CSF because RNA is very susceptible to nucleases. However, this may be overcome by incorporations of various modified RNA compounds into the aptamers, attachment of polyethylene glycol (PEGylation) to the aptamers, or mixing with RNase inhibitors. The SPC aptamers selected in this report have strong potential as new tools for development of a new SPC detection system.
